# Dioscin inhibits gastric tumor growth through regulating the expression level of lncRNA HOTAIR

**DOI:** 10.1186/s12906-016-1360-1

**Published:** 2016-09-30

**Authors:** Ting Ma, Rui-ping Wang, Xi Zou

**Affiliations:** 1 The Affiliated Hospital of Nanjing University of Chinese Medicine, Traditional Chinese Medicine of Changzhou Hospital, Changzhou, China; 2 Nanjing University of Chinese Medicine, Nanjing, China; 3 Jiangsu Province Hospital of TCM, Nanjing, China; 4 Department of Oncology, the Affiliated Hospital of Nanjing University of Chinese Medicine, Jiangsu Province Hospital of TCM, Nanjing, China

**Keywords:** HOTAIR, Gastric cancer, Dioscin, lncRNA

## Abstract

**Background:**

As a member of non-coding RNAs family, long non-coding RNAs’ functions in cancer needs to be further investigated. It has been indicated that the functions of Hox transcript antisense intergenic RNA (lncRNA: HOTAIR) include reprogramming chromatin organization and promoting tumor metastasis such as breast and colorectal tumor. The aim of this study is to investigate the functions of Hox in gastric cancer.

**Methods:**

In the present study, the expression level of HOTAIR was determined by quantitative reverse transcription polymerase chain reaction (qRT-PCR), 20 gastric cancer tissues and 20 normal tissues was included. All clinical data were analyzed retrospectively. The CCK-8 and colony formation assay was used to identify if the knockdown of HOTAIR have an influence on gastric cancer cell lines.

**Results:**

Compared with normal tissues, higher expression level of HOTAIR was found in gastric cancer tissues. Dioscin inhibits proliferation of the three gastric cancer cell lines and decrease HOTAIR expression.

**Conclusions:**

The expression of HOTAIR is up regulated in gastric cancer and gastric cancer cell lines, dioscin inhibits the proliferation of three gastric cancer cell lines and the anti-tumor effect of dioscin may partly depend on the down regulation of HOTAIR.

## Background

Gastric cancer is one of the most prevalent and deadly malignancies worldwide, with an estimated more than 950,000 new cases every year and 720,000 deaths reported in 2012 [1, 2]. With the advances in understanding of the ideology of the disease and therapeutic options, the 5-year mortality rate has only been slightly reduced over the last few decades. There is still a pressing need to identify new prognostic biomarkers and therapeutic targets for this disease [3].

As reviewed by Ling et al. [4] traditional Chinese medicine (TCM) can play a vital role in cancer prevention and treatment. The anticancer mechanisms of which involves inducing apoptosis, anti-proliferation, preventing tumor invasion and metastasis, and reducing resistance to chemotherapy [5]. More than co-administrate with other drugs to reduce side effects, TCM-derived drugs may also serve as gene therapy vehicles, therapeutic genes and synergistic therapeutic treatments [6, 7].

### An important class of non coding RNAs are the IncRNAs or long non coding RNAs

The functions of these RNAs involving vary aspects of genetics [8, 9]. Until HOTAIR was discovered, it is widely believed that lncRNAs mainly function in cis. Recent studies have demonstrated that lncRNAs are involved in the development of different types of cancer [10, 11], for example, metastasis associated lung adenocarcinoma transcript 1 (MALAT-1) in non small cell lung cancer and HOTAIR in breast cancer and colorectal cancer [12–14]. The expression level of genes related to cancer progression was regulated by HOTAIR via interacting with PCR2 complex [9]. However, the significance of HOTAIR expression in gastric cancer tissues from clinic has not been fully studied. In the present study, the expression level of HOTAIR in gastric cancer and adjacent normal tissues was detected.

Dioscin is an active ingredient identified in edible medicinal plants such as Dioscorea nipponica Makino and Dioscorea zingiberensis Wright [15]. It has been demonstrated that dioscin has anti-tumor [16] and anti-fungal activities. It has been known that Dioscin induce apoptosis and cell cycle arrest in gastric cancer cells [17]. Dioscin-induced apoptosis is mediated by multiple genes or proteins including caspase-9, caspase-3 and anti-apoptotic Bcl-2 protein [18, 19]. Dioscin could also induce apoptosis through elevating oxidative stress by downregulating peroxiredoxins, promoting ROS accumulation, inducing DNA damage, and activating mitochondrial signal pathways [20, 21]. However, the anticancer mechanisms of dioscin in human gastric cancer cells have not been delineated. The purpose of this study is to investigate the anticancer effects and molecular targets of dioscin on human gastric cancer cell lines including SGC-7901, MGC-803, and HGC-27. Our results show that the anticancer activities of dioscin against gastric cancer cells may partly depend on the down regulation of long non-coding RNA HOTAIR.

## Methods

### Ethics, consents and permissions

The protocol was approved by the Institutional Review Board at the Traditional Chinese Medicine of Changzhou Hospital (Changzhou, Jiangsu, China). An informed consent form was signed by each participant.

### Demographics

All participants were asked to answer 12 items relating to basic demographics: age, race, ethnicity, sex, education, marital, employment and insurance status, likelihood of insurance billing, household income, and self-reported height and weight. The answers were not scored and treated categorically in data analyses.

### Gastric cancer tissue and cell culture

Gastric cancer tissues were obtained from the Traditional Chinese medicine of Changzhou Hospital (Changzhou, Jiangsu, China). All tissues were immersed in RNA fixative after resection (TIANGEN, Beijing, China) and were deposited in −80 °C until use. The age range is between 48 and 70 years and the median is 56 years. The gastric cancer cell lines HGC-27, MGC803, and SGC7901 and normal gastric cell line GES-1 were purchased from the Type Culture Collection of the Chinese Academy of Sciences (Shanghai, China). All cell lines were cultured in RPMI1640 medium supplemented with 10 % fetal bovine serum (FBS), 1 % antibiotic-antimycotic solution, and maintained in a humidified incubator with 5 % CO_2_ at 37 °C.

### siRNA knockdown assay

HOTAIR-siRNA was performed as previously described [22, 23]. A total of 1.5 × 10^5^/well HGC-27, MGC803, SGC7901 or GES-1 were seeded into 6-well plates and were transfected with HOTAIR siRNAs 24 h later using RNAiMAX transfection reagents (Invitrogen, Shanghai, China) according to the instructions. The target sequence of HOTAIR siRNA was UUUUCUACCAGGUCGGUAC.

### RNA extraction and quantitative reverse transcription polymerase chain reaction

Total RNA was extracted as previously described [24]. The primers used in this study were HOTAIR: forward: 5′- GGTAGAAAAAGCAACCACGAAGC −3′ and reverse: 5′- ACATAAACCTCTGTCTGTGAGTGCC −3′; GAPDH: forward: 5′-AGGACTGGATAAGCAGGGCG −3′ and reverse: 5′-CTGGAACAGGGAGGAGCAGA −3′.

### MTT assay

Cells were plated into 96-well plates at a density of 2000 cells/well. The cells were incubated with different concentration of dioscin (Chengdu Ruifeng Biotechnology Company, Chengdu, China) (0 μmol, 10 μmol, 20 μmol, 30 μmol, 40 μmol, 60 μmol) for 48 h. Then the cells were incubated with 3-(4,5-dimethylthiazol-2-yl)-2,5-diphenyltetrazolium bromide (MTT) for 4 h at 37 °C. The absorbance at 490 nm was measured.

### Colony formation assay

Cells were seeded into 6-well plates at a density of 500 cells/well. Approximately 10 days later, the cells were fixed with methanol for 10 min and then stained with 0.5 % crystal violet in 20 % methanol. The number of colonies was counted and representative images were obtained.

### Statistical analysis

All data were shown as the mean. ± SD, and the experiments were repeated three times. One-way ANOVA was performed, and *P* < 0.05 was considered statistically significant.

## Results

### HOTAIR expression in clinical tissues, gastric cancer cell lines and noncancerous cell lines

**Fig. 1:**
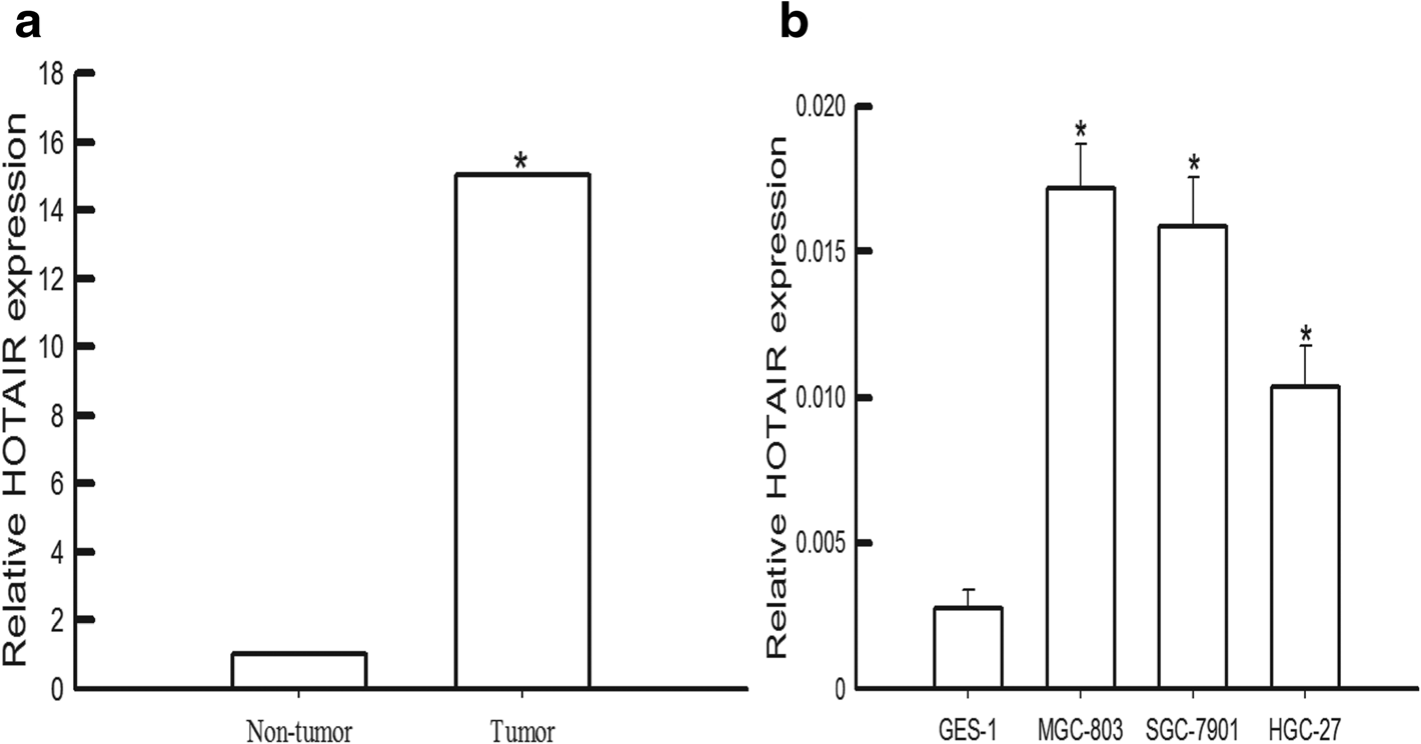
HOTAIR expression in gastric tumor tissues and gastric cancer cell lines was elevated. **a** HOTAIR expression was significantly higher in gastric cancer tissues than in non-cancerous tissues. Relative HOTAIR expression was determined using qRT-PCR with GAPDH as the internal control (number of patients was forty, *n* = 40). **b** HOTAIR expression was significantly higher in gastric cancer cell lines were higher than in normal gastric cell line. *P < 0.05 vs non-tumor tissues

The expression of HOTAIR between gastric cancer tissues and corresponding normal tissues was identified by qRT-PCR. As shown in Fig. 1, HOTAIR expression in gastric cancer tissues was more than 30-fold of that in normal tissues, suggesting that the expression of HOTAIR was upregulated in gastric cancer. HOTAIR expression in gastric cancer cell lines SGC-7901, MGC-803 and HGC-27 were significantly higher than that in noncancerous cancer cell line GES-1 (Fig. 1b).

### Effect of dioscin on gastric cancer cell lines and noncancerous cell line

**Fig. 2:**
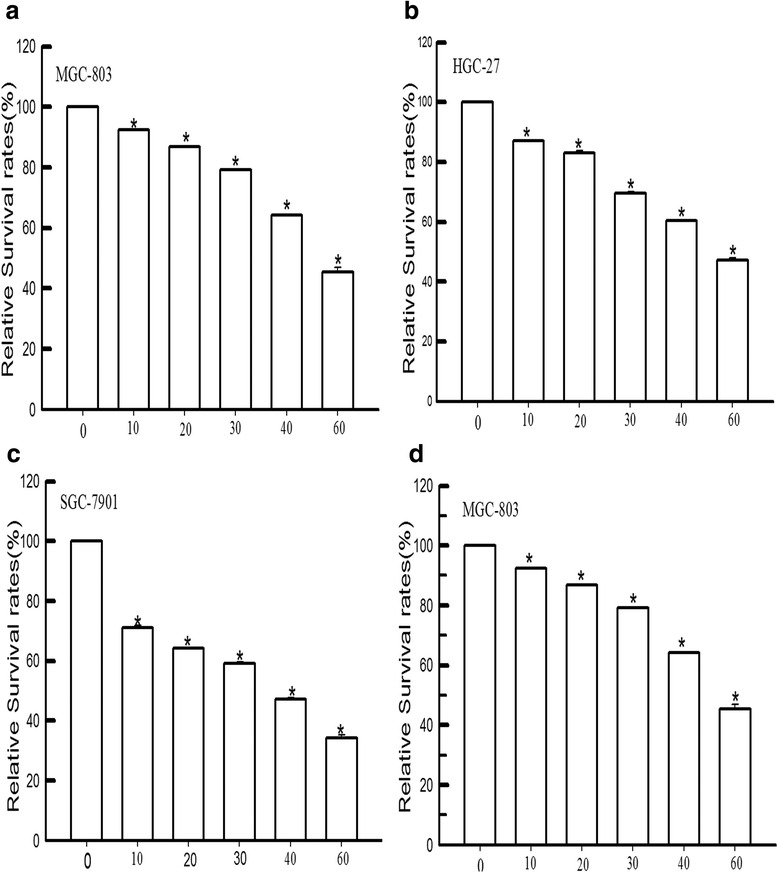
Dioscin inhibited gastric cancer cell proliferation in a dose-dependent manner. **a** the relative survival rates were markedly decreased with the dose of dioscin exceeding 10 μmol/L in MGC-803 cells. **b** the relative survival rates were markedly decreased with the dose of dioscin exceeding 10 μmol/L in HGC-27 cells. **c** the relative survival rates were markedly decreased with the dose of dioscin exceeding 10 μmol/L in SGC-7901 cells. **d** the relative survival rates were markedly decreased with the dose of dioscin exceeding 10 μmol/L in GES-1 cells. *P < 0.05, vs Control groups

We performed MTT analysis to assess the effect of dioscin on the proliferation of gastric cancer cells. The relative survival rates of gastric cancer cells were markedly decreased with doses of dioscin over 10 μmol/L(*P* < 0.05) (Fig. 2), suggesting that dioscin inhibited gastric cancer and noncancerous cell proliferation in a dose-dependent manner.

### HOTAIR knockdown or dioscin decrease cell proliferation in gastric cancer cells

**Fig. 3:**
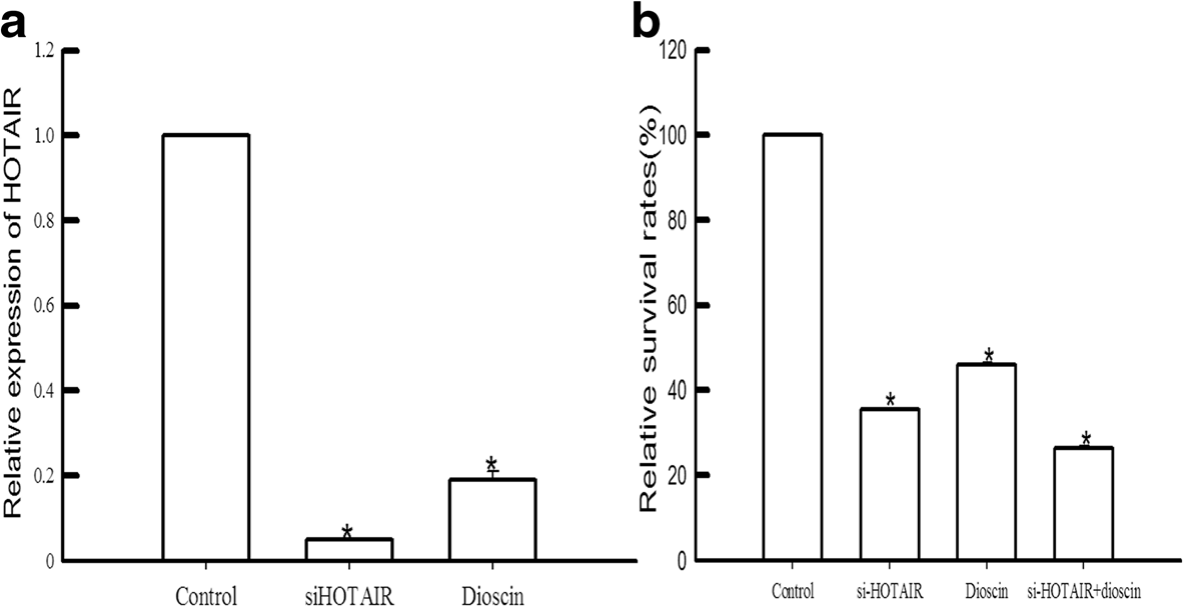
HOTAIR knockdown or dioscin decreased cell proliferation in gastric cancer cells. **a** relative expression of HOTAIR in MGC-803 cell transfected with HOTAIR siRNA or treated with dioscin. **b** The relative survival rates were markedly decreased with dioscin or HOTAIR siRNA in MGC-803 cells

**Fig. 4:**
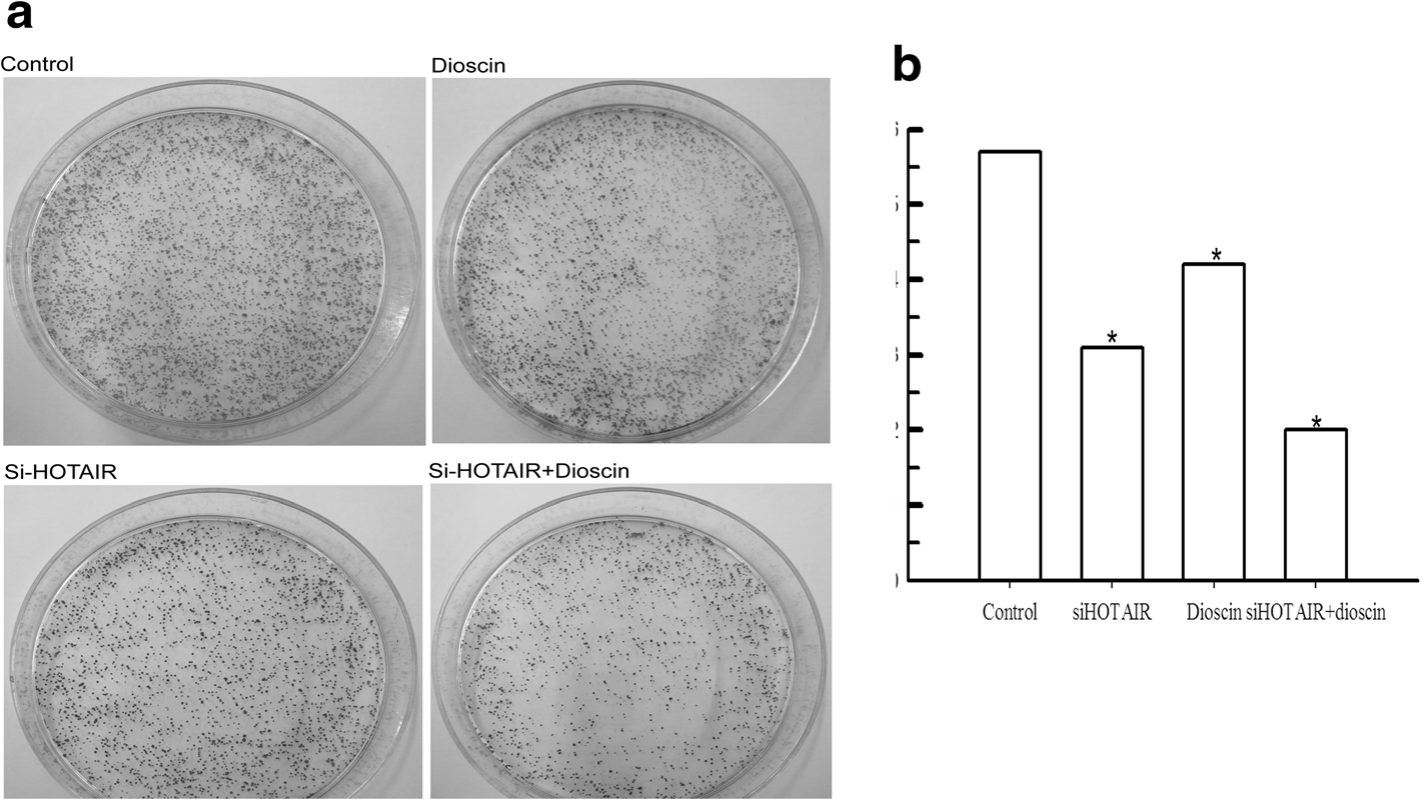
HOTAIR knockdown or dioscin decreased cell proliferation in gastric cancer cells. Knockdown cells were transfected with si-HOTAIR or treated with dioscin for 24 h and then plated in 6-cm dishes for 10 days. *P < 0.05, vs Control groups. **a** Colony formation assay, the cells were stained with crystal. **b** Number of colonies

Based on the results of Figs. 1 and 2, we choose gastric cell line MGC-803 and the concentration of dioscin at 56 μmol (Half Maximal Inhibitory Concentration) for further studies. To determine the function roles of HOTAIR and dioscin in gastric cancer, siRNA was used to silence HOTAIR expression, and we also found dioscin down regulated the expression of HOTAIR (Fig. 3a). MGC-803 cells were transfected with HOTAIR siRNA or treated with dioscin for 48 h, then their proliferation was examined using MTT and colony formation assays. As shown in Fig. 4, both HOTAIR siRNA and dioscin could significantly inhibit MGC-803 cells proliferation. The same effect was observed in the colony formation assays.

## Discussion

The expression level of HOTAIR was markedly up regulated in many cancers, which play an important role in multiple biological progress of tumor development [25]. Our data shown the expression level of HOTAIR was drastically higher in gastric cancer tissues and gastric cancer cell lines (MGC-801, SGC-7901 and HGC-27). These data were consisted with previous studies which found the expression of HOTAIR was higher in cancer lesions than in adjacent normal tissues in gastric cancer [26, 27].

Among the three cell line, the expression level of HOTAIR in MGC-801 was the highest which was used for further MTT and colony formation assay. The data indicated that HOTAIR involved in the proliferation process and colony formation of MGC-801 (shown in Figs. 3B and 4E). It has been shown that HOTAIR expressed highly in high-risk gastrointestinal stoma tumors, and the high expression levels were related to tumor metastasis [28]. Moreover, recent study indicated that HOTAIR function as lymphatic nodes metastasis and survival predictor [27]. Taken together, HOTAIR played an important role in the pathological process of gastric cancer.

It has been known that dioscin inhibits tumor growth through multiple pathways. In colon cancer, dioscin efficiently inhibited VEGFR2 and AKT/MAPK pathways mediated angiogenesis [29]. Dioscin-induced breast cancer cell death via AIF-facilitated caspase-independent pathway and the down regulation of anti-apoptotic proteins as Bcl-2, cIAP-1, and Mcl-1 [30]. Previous study showed that the activation of the mitrochondrial pathway was associated with antipoliferative effect of dioscin in gastric cancer [31]. Here we showed that dioscin may inhibit the proliferation of gastric cancer through decreasing the expression level of HOTAIR. Further studies are needed to illustrate how dioscin inhibits HOTAIR expression.

## Conclusions

In conclusion, these data showed that the expression of HOTAIR was up regulated in gastric cancer and gastric cancer cell lines. Dioscin inhibited the expression of HOTAIR and the proliferation rate of gastric cancer cells, indicating that the antitumor effect of dioscin may partly depend on its down regulation of HOTAIR.

## Abbreviations

FBS: Fetal bovine serum
HOTAIR: HOX transcript antisense RNA
lncRNA: long non-coding RNAs
MALAT-1: Metastasis associated lung adenocarcinoma transcript 1
MTT: 3-(4,5-dimethylthiazol-2-yl)-2,5-diphenyltetrazolium bromide
TCM: Traditional Chinese medicine
